# American Medical Association

**Published:** 1867-06

**Authors:** 


### *American Medical Association.*

This body convened in Cincinnati, Tuesday, May 7th. The President, Dr. H. F. Askew, of Delaware, in the Chair. Welcoming address by Dr. J. A. Murphy, of Cincinnati, Chairman of Committee of Arrangements. President Askew's address is characterized as "pertinent, important, and comprehensive." Several of the Committees reported, more did not, and several were ultimately discharged. The inevitable question of rank of naval surgeons was resolved upon. The women-doctor innovation frowned upon. A circular, adopted by the delegates of several medical colleges, was read, and debate upon it denied. The Surgeon-General was censured, and Prof. S. T. Gross elected President. Are not the remainder of the transactions written for the July No. of the JOURNAL?

